# Synchronous advanced gastric adenocarcinoma and advanced esophageal squamous cell carcinoma

**DOI:** 10.1590/S1516-31802002000100008

**Published:** 2002-01-02

**Authors:** Fernando Augusto Mardiros Herbella, Laércio Gomes Lourenço, José Carlos Del Grande, Rafael Possik, Chibly Michel Haddad

**Keywords:** Esophagus, Stomach, Cancer, Synchronous, Esôfago, Estômago, Câncer, Sincrônico

## Abstract

**CONTEXT:**

Synchronous associations of esophageal and gastric cancers are not a common finding, especially with differing histological types and both tumors in advanced forms. A case with such an association is presented, in which an unusual therapy was proposed: palliative gastrectomy and esophageal intubation.

**CASE REPORT:**

A 75-year-old white man was referred to our service complaining of malaise and weight loss for one year and dysphagia and vomiting for 2 months. The patient had sought out medical consultation as a result of the latter two complaints.

## RESUMO

**CONTEXTO:**

A associação sincronicamente de cânceres do esôfago e estômago não é um achado comum, especialmente com tipos histológicos diferentes e ambos os tumores avançados. Um caso com essa associação é relatado e proposta uma terapia: gastrectomia paliativa e prótese esofágica.

**RELATO DE CASO:**

Um paciente do sexo masculino, branco, de 75 anos, foi referido ao nosso serviço queixando-se de mal-estar e perda ponderal por um ano e vômitos por dois meses, motivo pelo qual procurou ajuda médica.

## INTRODUCTION

Esophageal and gastric cancer share some putative risk factors, including diet, low socioeconomic status, age, alcohol and tobacco use, nitrites and nitrates. However, associations between the two cancers are a rare presentation, especially when the histological types differ and both neoplasms are in advanced forms.

The authors describe a case with such an association.

## CASE REPORT

A 75-year-old white man was referred to our service complaining of malaise and weight loss for one year and dysphagia and vomiting for 2 months. The patient had sought out medical consultation as a result of the latter two complaints. He was an alcoholic and heavy smoker, suffering from chronic obstructive pulmonary disease. Endoscopy revealed an esophageal tumor in the middle third of the thoracic esophagus measuring 8 centimeters and a Borrmann III antral tumor measuring 5 centimeters. Histological analysis revealed an esophageal squamous cell carcinoma and a gastric adenocarcinoma.

**Figure 1:**
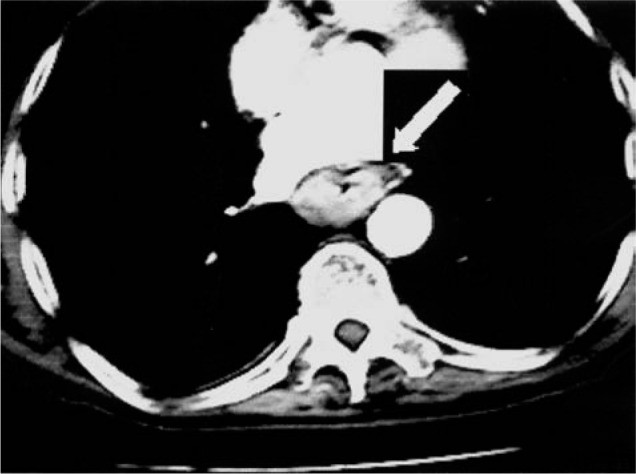
Thoracic tomography. Arrow shows esophageal tumor.

**Figure 2:**
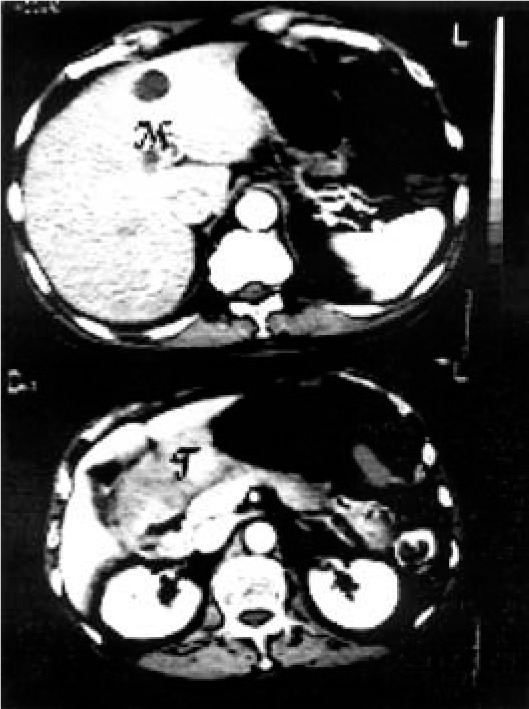
Abdominal tomography. T - gastric tumor, M - hepatic metastasis.

Preoperative staging showed normal bronchoscopy; an esophageal tumor without a cleavage plane and with the aorta on thoracic tomography (CT) (Figure 1); an antral tumor and multiple hepatic metastases on abdominal CT (Figure 2).

In view of the presence of both of these advanced obstructive diseases and the patient's pulmonary condition, it was decided to resect the gastric carcinoma and intubate the esophageal tumor.

A subtotal gastrectomy was performed and a Malafaia tube was inserted during the surgery, using the pull technique. No intraoperative complications occurred.

During the course of the postoperative period, pneumonia was detected. However, no surgical complications were present.

## DISCUSSION

The association described is rare. Some cases have been reported, especially by authors from Japan, where the number of patients operated due to gastric and esophageal carcinoma is high.

The value in reporting this case resides in the way it highlights the problems in digestive tract reconstruction after esophagectomy. It shows the need for adequate evaluation of the stomach in esophageal neoplasms and the need for the right therapeutic choice.

Alimentary tract restoration can be accomplished using the remaining stomach,^1^ depending on the location of the gastric tumor. However, the colon^1,2^ is frequently the choice for the esophageal substitution. Obviously, this problem is also found in metachronous gastric and esophageal cancers, a more frequent association.

There is a clear need for evaluating the stomach even in obstructive esophageal neoplasms and this has already been discussed.^1,3^ Gastric tumors not detected preoperatively have been described in 28 to 40% of cases of this chance association.^1,4^

It is evident that in non-advanced cases the right treatment is esophagogastrectomy.^1^ The options are limited when advanced cases in patients with poor clinical conditions are considered. Palliative non-surgical management can be chosen,^3^ or an endoscopic re-section or palliation can be achieved for esophageal or gastric tumors.^3^ Although palliative esophagogastrectomy can be successfully performed,^2^ we believe it is a high risk procedure. However, this is not true for a palliative gastrectomy.

Our conduct seemed reasonable considering that palliation for dysphagia was achieved with low morbidity-mortality.
